# On-Chip Isoniazid Exposure of *Mycobacterium smegmatis* Penicillin-Binding Protein (PBP) Mutant Using Time-Lapse Fluorescent Microscopy

**DOI:** 10.3390/mi9110561

**Published:** 2018-10-31

**Authors:** Meltem Elitas

**Affiliations:** Faculty of Engineering and Natural Sciences, Sabanci University, Tuzla, 34956 Istanbul, Turkey; melitas@sabanciuniv.edu; Tel.: +90-538-810-2930

**Keywords:** penicillin-binding protein (PBP), microfluidics, antibiotics, *M. smegmatis*, single-cell resolution, microscopy

## Abstract

Antibiotic resistance has been one of the biggest threats to global health. Despite the available prevention and control strategies and efforts in developing new antibiotics, the need remains for effective approaches against antibiotic resistance. Efficient strategies to cope with antimicrobial resistance require a quantitative and deeper understanding of microbial behavior, which can be obtained using different techniques to provide the missing pieces of the current antibiotic-resistance puzzle. Microfluidic-microscopy techniques are among the most promising methods that contribute modernization of traditional assays in microbiology. They provide monitoring and manipulation of cells at micro-scale volumes. Here, we combined population-level, culture-based assays with single-cell resolution, microfluidic-microscopy systems to investigate isoniazid response of *Mycobacterium smegmatis* penicillin-binding protein (PBP) mutant. This mutant exhibited normal growth in plain medium and sensitivity to stress responses when treated with thermal stress (45 °C), detergent stress (0.1% sodium dodecyl sulfate), acid stress (pH 4.5), and nutrient starvation (1XPBS). The impact of *msm0031* transposon insertion on drug-mediated killing was determined for isoniazid (INH, 50 µg/mL), rifampicin (RIF, 200 µg/mL), ethionamide (ETH, 200 µg/mL), and ethambutol (EMB, 5 µg/mL). The PBP mutant demonstrated remarkable isoniazid-killing phenotype in batch culture. Therefore, we hypothesized that single-cell analysis will show increased lysis kinetics and fewer intact cells after drug treatment. However, the single-cell analysis data showed that upon isoniazid exposure, the percentage of the intact PBP mutant cells was 24%, while the percentage of the intact wild-type cells was 4.6%. The PBP mutant cells exhibited decreased cell-lysis profile. Therefore, the traditional culture-based assays were not sufficient to provide insights about the subpopulation of viable but non-culture cells. Consequently, we need more adequate tools to be able to comprehend and fight the antibiotic resistance of bacteria.

## 1. Introduction

Antibiotic resistance remains one of the biggest threats to global health in spite of being an old fight for humankind. Better prevention and control plans and effective strategies against antibiotic resistance are needed [1]. The World Health Organization (WHO) categorizes emergence and spread of antimicrobial resistance as a high priority action, and aims to ensure its prevention and treatment with effective and safe medicine while developing new antibiotics or alternative medicine to antibiotics [2,3]. Efficient strategies to manage antimicrobial resistance require a quantitative and deeper understanding of microbial behavior. Combining the qualities of different techniques will provide development of novel approaches for antibiotic-resistance problems.

From Antonie van Leeuwenhoek to the 2000s, observation and investigation of microorganisms and development of antibiotics mostly relied on population-level assays, known as “bulk assays” [4]. The majority of the laboratories in academia, clinics, or pharmaceutical companies has continued to actively use these tools and methods. However, significant effort has been recently dedicated to increase the resolution of culture-based assays via the contributions of scientists from diverse disciplines, ranging from engineering, physics, statistics, medicine, and chemistry [5,6,7,8]. Our aim is to better understand microbial heterogeneity and antibiotic responses, not only during discrete physiochemical interactions at discrete-time points, but also to characterize their actions as dynamic, continuous time events while mimicking their pathophysiological conditions, such as microenvironments for biofilms [9]. Using microfabricated tools, scientists reported the importance of the microenvironment on motility, distribution and dynamics of bacterial culture [10,11,12,13]. In contrast to statically culturing cells in flasks, bacteria are cultured in microchannels, fed by a continuous medium flow where the antibiotic can be added into the medium, and the antibiotic response of the cells can be dynamically observed at single-cell resolution [10]. Therefore, these technologies have great potential for observing and quantifying the microenvironmental conditions that shape the survival strategies of bacteria, such as biofilms. Biofilms are strictly regulated by their surrounding microenvironments [14,15,16]; these might be able to be tightly controlled using microfluidic devices. Moreover, microfluidic cell culture systems have provided great potential for culturing currently viable but non-culturable bacteria (VBNCs) in laboratories, while discovering the mechanisms behind their phenotype [11]. Pioneering work by Dr. Austin and his co-workers has combined theoretical and experimental methods to unearth the cryptic complexity of bacterial evolution in studying the antibiotic response of the cells in dynamic, spatially heterogeneous microenvironments [17,18,19]. Dr. Ackermann and his group presented novel data about molecular microbial ecology using time-lapse microscopy and single-cell analysis [19,20,21,22,23]. From the fundamental side, these studies provide an ability to culture microbes in dynamic environments to reveal their role in the environment, ecology, and nutrient cycle. From the applied side, understanding of the underlying molecular mechanisms they develop to adapt to different stresses will be transferable to benefits for health, ecology and science. Uncovering the mystery of adaptation strategies to survive in the microbial word requires exploring bacteria in their complex, natural microenvironments; otherwise, impaired or underestimated results will mask their variability, and avoid conditions that cause antibiotic-resistant infections. Therefore, single-cell level and culture-based assays are complementary pieces of the current state-of-the-art.

In this study, we investigate the stress response of *Mycobacterium smegmatis* (*M. smegmatis*) transposon mutant, *msm0031::Tn*, using microfluidics and bulk assays. *M. smegmatis* is a rod-shaped bacterium with a complex cell wall [24,25]. It is a model organism for human pathogen, *Mycobacterium tuberculosis* (*M. tuberculosis*). However, it is a non-pathogen, fast-growing organism that requires a biosafety level 1 laboratory. It shares more than 2000 homologous genes with *M. tuberculosis* [26,27]. The product of the *msm0031* gene is a penicillin-binding protein (PBP) required for cell division and involved in cell wall formation. The *M. smegmatis msm0031::Tn* mutant has remarkable isoniazid-killing phenotype both at population and single-cell levels. 

PBPs are sets of membrane-bound enzymes, enrolled in glycan chain assembly and peptide cross-linking of bacterial peptidoglycan. The underlying beta-lactam resistance mechanism relies on modification of the PBPs in terms of copy number or affinity towards beta-lactams. Contribution of PBP mutations has been continuously investigated in various bacterial species regarding the development of tolerance to several antibiotics, including penicillin [28]. Here, we studied the influence of the PBP mutation on the isoniazid (INH)-killing kinetics of *M. smegmatis* using the method that was firstly reported by Wakamato et al. to show that the persistence of antibiotic stressed mycobacteria is dynamic [10]. Next, we used this approach to investigate the INH-killing profile of the NADH (reduced form of Nicotinamide adenine dinucleotide) pyrophosphate mutant of *M. smegmatis* [29]. Our findings reported that the NADH pyrophosphate mutant cells had a low level of cell division and a high level of cell lysis, and that they were incapable of regrowth upon INH removal. Contrary to our microfluidic-based approaches, in literature most mycobacterial studies have investigated PBPs using transposon mutants with specific phenotypes in batch culture [30]. Patru and Pavelka investigated the role of PonA2 and PonA3 in mycobacterial peptidoglycan biology. They reported that ΔPonA2 strains have decreased susceptibility to the beta-lactams ampicillin and ceftriaxone, increased susceptibility to rifampin (RIF), and no susceptibility difference for isoniazid (INH), ethambutol (EMB), vancomycin, or imipenem. ΔPonA3 strains did not affect the antibiotic susceptibility. PonA2 has a role in adaptation to survival and these cells have spherical morphology due to weakened cell wall integrity. Bansal et al. characterized putative DD-carboxypeptidase (DD-CPase) via cloning *MSMEG_2433* gene in *Escherichia coli* (*E.coli*) and showed that *MSMEG_2433* is both a DD-CPase and beta-lactamase [31]. Enany and co-workers presented the role of mycobacterial DNA-binding protein 1, which controls suppression of DNA synthesis, respiration, and fatty acid synthesis, and protection against reactive oxygen species, so prevents rapid cell death in the stationary phase and provides long-term survival of mycobacteria [32]. Moreover, Flores, Parsons and Pavelka performed transposon mutagenesis of the beta-lactamase mutants of *M. tuberculosis* and *M. smegmatis* and screened for cephalosporin and ceftriaxone hypersusceptible mutants. Their results provided unknown proteins that are involved in peptidoglycan biosynthesis, cell division, or other cell envelope processes [33]. Kieser et al. performed whole-genome transposon mutagenesis screens in *M. tuberculosis* deleted for PonA1, PonA2, and LdtB, major peptidoglycan enzymes. They verified that *M. tuberculosis* uses alternative pathways to produce peptidoglycan; either PonA1 or PonA2, which genetically interact with LdtB, is required for *M. tuberculosis* growth that also provides differential susceptibility to antibiotics [34].

To our knowledge, the *msm0031::Tn* mutant has not been studied in detail using both culture-based and microfluidic-microscopy-based assays. Our findings present the effect of the *msm0031* gene in increased INH-susceptibility. Our single-cell analysis complements the data obtained from culture-based assays while challenging the definition of death for bacteria.

## 2. Materials and Methods

### 2.1. Growth of the Cells

*M. smegmatis* cells were grown in standard Middlebrook 7H9 medium (BD/Difco) containing 0.085% NaCl, 0.5% albumin, 0.2% glucose, 0.5% glycerol, and 0.05% Tween-80 at 37 °C with shaking at 200 rpm [35]. Optical density of the cells (absorbance at 600 nm) was measured to determine the growth of the cells via a spectrophotometer (Thermo Scientific biomate 5, Waltham, MA, USA). 

### 2.2. Antibiotic Responses

Agar plates were prepared with 15 g/L Bacto agar (Sigma-Aldrich, St. Louis, MO, USA) and 15.5 g/L Luria Miller broth base (Sigma-Aldrich) in water. *M. smegmatis* cells were grown to OD_600_ of 0.5–1 and diluted to OD_600_ of 0.05, corresponding to ~10^7^ CFU/mL, in fresh 7H9 medium. Serial 10-fold dilutions were made using 100-µL cultures and 900-µL 1X PBS—0.025% Tween solutions in 5-mL polypropylene tubes. Appropriate dilutions of 100 µL were plated onto agar-based media to ensure that the serial dilutions would give at least one countable plate. Unless otherwise specified, antibiotics were used at the following concentrations: INH: 50 µg/mL, RIF: 200 µg/mL, ETH: 200 µg/mL, EMB: 5 µg/mL. Then, plates were incubated at 37 °C and colonies were enumerated on day 4.

### 2.3. Environmental Stresses

Sensitivity of the *msm0031::Tn* mutant and wild-type cells to thermal stress (45 °C), the detergent sodium dodecyl sulphate (0.1%. SDS), acidic pH (pH 4.5), and nutrient starvation by incubation of cells in phosphate-buffered saline (PBS) was determined [35]. 

### 2.4. Minimum Inhibitory Concentration (MIC)

*M. smegmatis* cells were grown to OD_600_ of 0.5–1 and diluted to OD_600_ of 0.05 in fresh 7H9 medium. Then 10-fold serial dilutions were plated on Luria broth (LB) agar solid plates containing various concentrations of INH. MIC was defined as the lowest concentration of drug required seeing minimum bacterial colony-forming unit (CFU).

### 2.5. Transposon (Tn) Mutagenesis and Identification of the msm0031::Tn Mutant

The fMycoMarT7 transposon donor phagemid (provided by Eric Rubin, Harvard School of Public Health), which carries a selectable marker for kanamycin resistance, was used to mutagenize a strain of *M. smegmatis* mc^2^155 [32]. For identification of the *msm0031::Tn* mutant, genomic DNA was extracted using lysozyme, proteinase K digestion, cetrimide saline, and chloroform extraction [33]. Then, genomic DNA was digested with BamHI (New England BioLabs, NEB, Ipswich, MA, USA), the digested DNA was ligated with T4 ligase (NEB) and transformed into Pir1 competent *E. coli* cells (Invitrogen, Carlsbad, CA, USA). Kanamycin-resistant colonies were selected; plasmid DNA was isolated, and sequenced using the primer cttctgagcgggactctgggg (Supplementary Materials, Figures S1–S3) [29,36].

### 2.6. Microfluidic Chip and Live-Cell Imaging

**Figure 1:**
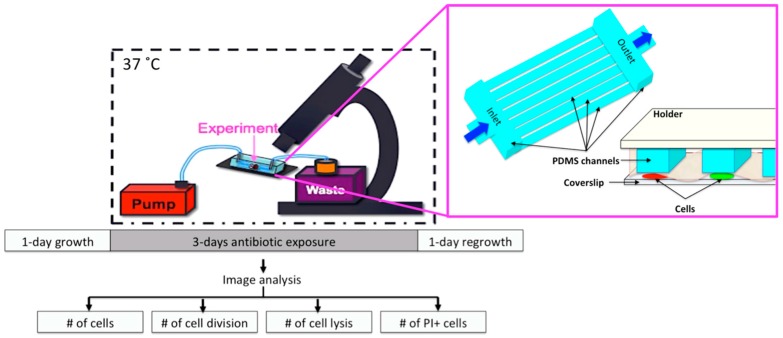
Schematics of the on-chip experiments and PDMS microfluidic device.

The microfluidic chip provided growth of the cells between the coverslip (#1) and the semipermeable membrane (Spectrum Lab, MWCO:8, Rancho Dominguez, CA, USA), which was under the Polydimethylsiloxane (PDMS, Sylgard 184) microfluidic channels with the same diameter (22 mm) of the coverslip, Figure 1. The microfluidic network considered a series of parallel channels in between the inlet and outlet with a 50 µm × 50 µm height and width [10]. Wakamato et al. presented the design and fabrication of the device [10]. Continuous medium flow (25 µL/min) was provided using standard, silicone tubing (ID: 0.076 cm, OD: 0.165 cm, HelixMark, Würselen, Germany) and a syringe pump (World Precision Instruments, Sarasota, FL, USA). Medium switching was very practical for on-chip drug exposures [10]. Thanks to our microfluidic network, the cells were sandwiched between the membrane and coverslip, and the microchannels were on top of the membrane; therefore, the depth of the microchannels did not introduce any problems, such as cell-to-cell overlapping or focusing of cells (Figure 1). 

Moreover, the microfluidic device enabled the monitoring of single-cells during the pre-antibiotic, antibiotic, and post-antibiotic periods for 72 h, which traditional methods cannot provide. Live-cell imaging was performed using an inverted, automated, time-lapse fluorescent microscope (Olympus IX75, Hamburg, Germany) equipped with a Hamamatsu ORCA-AG CCD camera. Using 100× oil-immersion objective (UPLFLN) images were acquired on phase and fluorescent channels (TRIS-red, GFP-green, exposure: 150 µs). The microfluidic device loaded with cells was mounted on the microscope inside a temperature-controlled chamber (37 °C).

### 2.7. Image Analysis

Time-lapse fluorescent movies were analyzed using cell counter plugin in ImageJ. The numbers of lysis and division events were manually counted using the cell counter plugin for every 4-h time frame. When the cells lysed, they lost their fluorescence. When the cells divided, their siblings appeared and expressed fluorescence. 

In order to ensure the division and lysis events, the movies, obtained via merging the fluorescence and phase contrast images, were analyzed. The figures were plotted in Prism4. 

### 2.8. Statistical Analysis 

The Student’s unpaired *t*-test (two-tailed) was used to assess statistical significance of pairwise comparisons. *p*-values were calculated using online software, GraphPad QuickCalcs. *p*-values < 0.05 present statistically significant differences. Three independent experiments were performed and used to determine the *p*-values.

## 3. Results

### 3.1. Batch-Cultue Behavior of the msm0031 Transposon Mutant and Wild-Type M. smegmatis Cells

Growth and stress responses of the cells, particularly to antibiotics, was conducted using population-level, batch-culture assays. 

**Figure 2:**
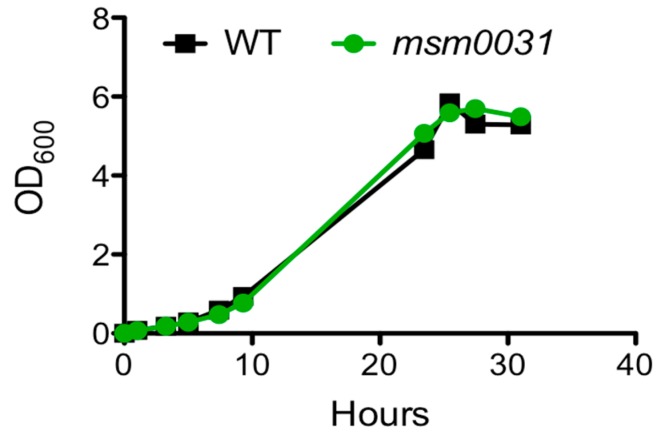
Growth of the *msm0031::Tn* mutant and wild-type (WT) *M. smegmatis* cells in batch culture.

Growth was determined by monitoring optical absorbance of the *msm0031* transposon mutant and wild-type (WT) cells in standard 7H9 medium. The *msm0031* transposon mutant was capable of growth with WT kinetics (Figure 2; Supplementary Materials, Figures S4 and S6). 

### 3.2. Drug Specificity

**Figure 3:**
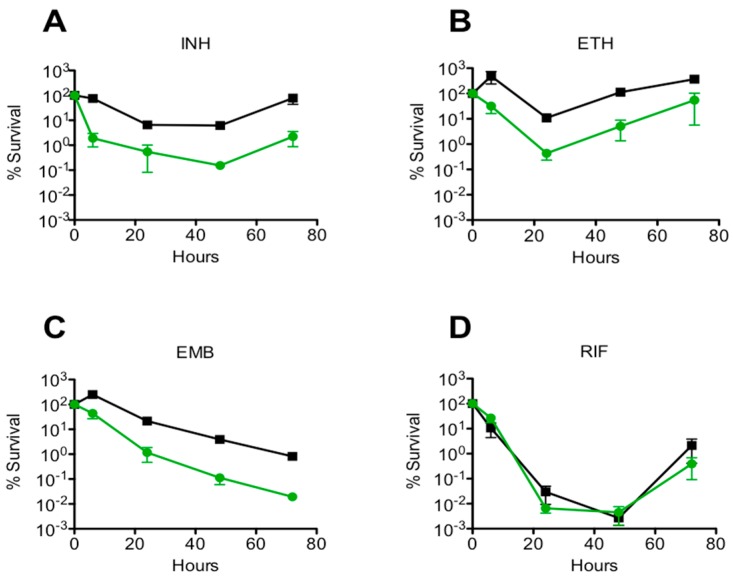
Batch culture drug-killing assay for the *msm0031* transposon mutant (green circles) and wild-type *M. smegmatis* cells (black squares). (**A**) INH, 50 µg/mL; (**B**) ETH, 200 µg/mL; (**C**) EMB, 5 µg/mL; and, (**D**) RIF, 200 µg/mL. Results are the means and standard errors from 3 independent experiments.

The impact of *msm0031* transposon insertion on drug-mediated killing was determined for INH, ETH, EMB, and RIF, Figure 3.

Since the influence of INH for the PBP mutant was investigated, the MIC value of the *msm0031* transposon mutant for INH was determined as 3.125 µg/mL. The INH-MIC value of the wild-type *M. smegmatis* cells was measured as 3.125 µg/mL. 

The drug specificity of the *msm0031* transposon mutant was tested to determine whether the mutant is phenotypically tolerant to a wide range of antibiotics (Supplementary Materials, Figures S5–S7). Comparison of persistence phenotype rates was defined as the fractional survival ratio (FSR). The FSR values were calculated as percentage survival of mutant cells divided by percentage survival of wild-type cells after 48 h of exposure to INH, RIF, ETH, and EMB [32]. FSR > 1 persisted better than WT; FSR < 1 showed decreased persistence compared to wild-type. *p*-values were calculated using the Student’s unpaired *t*-test. *p*-values < 0.05 were statistically significant. Mean values and standard errors were calculated from data obtained in three independent experiments (Table 1).

### 3.3. Stress Responses

**Figure 4:**
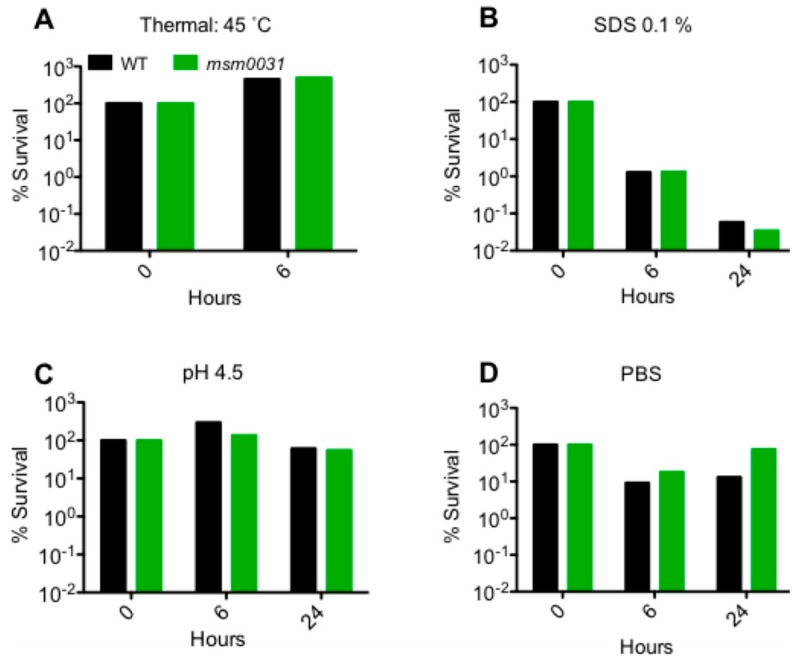
Sensitivity of the *msm0031::Tn* mutant and wild-type *M. smegmatis* cells to (**A**) 45 °C; (**B**) 0.1% sodium dodecyl sulfate; (**C**) pH 4.5; and, (**D**) PBS (1X).

The responses of the *msm0031::Tn* mutant to other external stresses, including thermal stress (elevated temperatures of 45 °C), detergent stress (0.1% sodium dodecyl sulfate), acid stress (pH 4.5), and nutrient starvation (incubation in phosphate-buffered saline, PBS 1X), were investigated (Figure 4) [37].

### 3.4. On-Chip Behavior of the msm0031 Transposon Mutant and Wild-Type M. smegmatis Cells

On-chip experiments allowed us to simultaneously observe single cells in a mixture of both wild-type cells (green fluorescent) and the *msm0031* transposon mutant cells (red fluorescent) while excluding the possibility that differences in the growth environment might be the cause of the observed phenotypes in previous assays (Supplementary Materials, Figure S4). Figure 1 illustrates how on-chip experiments were conducted and image analysis was performed. 

**Figure 5:**
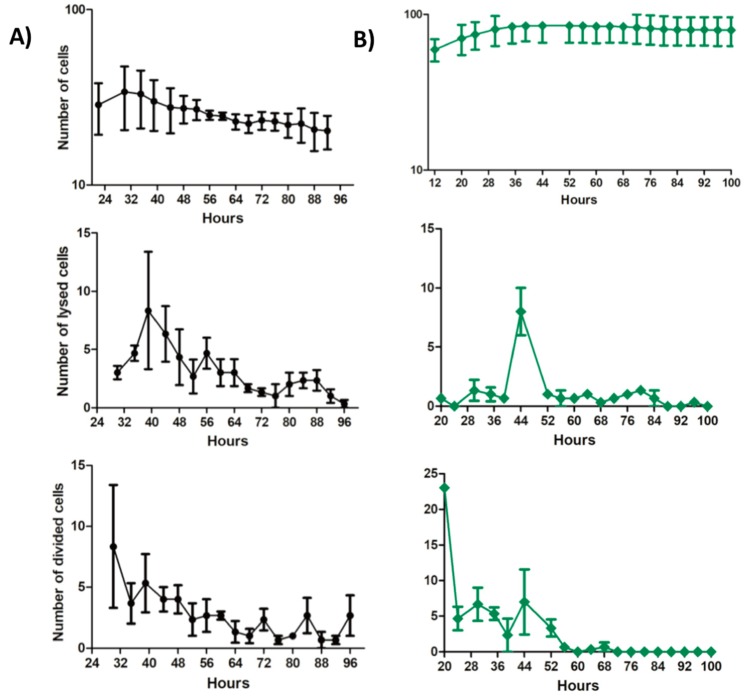
Quantitative single-cell analysis using on-chip-microscopy images of wild-type *M. smegmatis* (**A**) and the *msm0031::Tn* mutant (**B**) during 72 h of INH (50 µg/mL) exposure. The first panel illustrates the cumulative behaviour of the cells as a composite of lysis and division events. The second panel shows the number of cells that lysed during each successive time interval. The third panel presents the number of cells that divided in each successive time interval. The data present the mean and standard errors of three points in the microfluidic device.

The on-chip INH-killing experiment consisted of three steps: growth of the cells in standard 7H9 medium; three-day INH exposure for differential killing; and drug withdrawal to observe recovery from the antibiotic treatment [29]. Observation of the cells was achieved using live-cell imaging on the fluorescent microscopy (Supplementary Movie). Figure 5 demonstrates the on-chip behavior of the *msm0031::Tn* mutant and wild-type cells at single-cell resolution.

**Figure 6:**
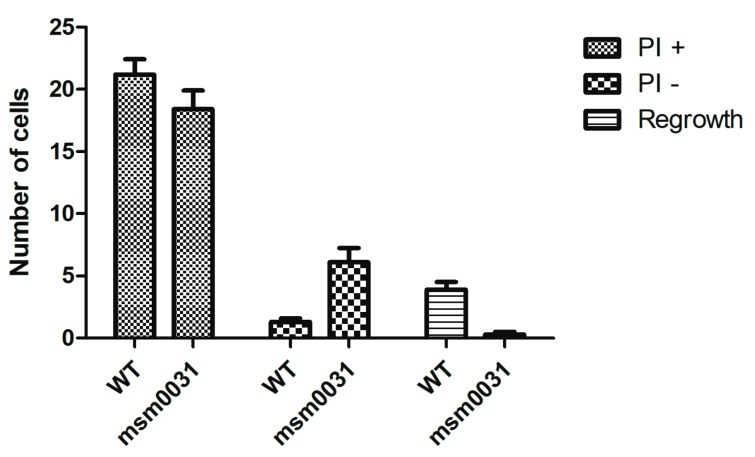
Phenotypic subpopulations of INH-exposed *M. smegmatis* wild-type and the *msm0031::Tn* mutant cells. The mean values show the number of cells, the error bars show the standard errors of ten different points in the microfluidic device. PI stands for propidium iodide staining. PI +: PI stained cells, PI-: PI-free cells.

After drug withdrawal, an end-point staining was performed with propidium iodide (PI). In order to quantify the PI-positive, PI-negative and regrowth subpopulations of the cells, we used wild-type cells expressing green fluorescent protein, *msm0031* transposon mutant cells expressing no flouresecent protein, and PI (red dye). For *M. smegmatis* wild-type cells, the number of intact cells following INH exposure was 512; 417 of these were PI-positive, 24 were PI-negative, one cell lysed, and 70 resumed growth after INH washout. Regarding the *M. smegmatis msm0031::Tn* mutant, the number of intact cells following INH exposure was 795; 605 of these were PI-positive, 190 were PI-negative, 4 resumed growth, and none of the cells lysed after INH washout. Figure 6 shows the comparison of WT and the *msm0031* transposon mutant behavior at ten different points in the microfluidic device. 

## 4. Discussion

This work presented the behaviour of *M. smegmatis msm0031* transposon mutant and wild-type cells both at single-cell level in conjunction with a microfluidic-microscopy system and at population-level using traditional, batch-culture assays. Since INH targets the mycobacterial envelope [29] and the *msm0031* gene is a PBP involved in cell wall formation and cell division [28,29,30,31], our investigations mainly focused on the INH-killing profile of the *M. smegmatis msm0031* transposon mutant. 

In our studies, we obtained the *M. smegmatis msm0031* mutant using transposon mutagenesis, which is one of the common genetic tools to obtain the specific mycobacterium phenotypes [33,34]. In literature, most of the PBP-related studies were performed using culture-based assays. These studies mainly focused on either antibiotic action mechanisms or bacterial peptidoglycan biosynthesis concepts. For the first concept, the antibiotic-killing profile of the PBP mutants was mostly questioned to understand how beta-lactam antibiotics work and how mycobacteria develops resistance to beta-lactams, such as penicillin [28], ampicillin, ceftriaxone [30], vancomycin, imipenem [31], cephalosporin, and ceftriaxone [32]. Among these studies, only Pature and Pavelka’s research focused on INH susceptibility of the ΔPonA2 strains in addition to beta-lactams while studying the role of the PonA2 and PonA3 genes in mycobacterial peptidoglycan formation [30]. The hypothesis of PBP enrollment in the cell envelope and cell division processes was mostly reported for the PonA1, PonA2 and LdtB proteins [33]. Next, they focused on purification and biochemical characterization of PBP proteins of mycobacterium. Patru and Pavelka’s study reported the relationship between the spherical morphology of the cells and their antibiotic susceptibility via PBP proteins. 

Here, we implemented single-cell level, microfluidic-microscopy and population-level, culture-based assays to investigate *M. smegmatis* transposon mutant, *msm0031*. First, we confirmed that there was no growth phenotype for the *msm0031* transposon mutant. Next, we obtained the *msm0031* deletion mutant. The *msm0031* deletion strain presented hindered growth, defective cell separation, higher transcript levels and a cell area twice as large compared to wild-type cells (Supplementary Materials, Figures S3–S5). Then, we performed batch-culture drug susceptibility assays using INH, ETH, EMB, and RIF [38]. Among them, RIF killed both the PBP mutant and wild type cells until 48 h; afterwards, rifampicin degraded and the cells regrew (Figure 3) [39,40,41]. The PBP mutant presented enhanced killing profile for INH, ETH, and EMB; these drugs targets cell wall components (Figure 3, Table 1). In addition, we determined the sensitivity of the *msm0031* transposon mutant to thermal stress, detergent, acidic pH, and nutrient starvations to confirm the INH-killing profile of the *msm0031* transposon mutant was not mainly due to impaired cell wall formation (Figure 4). Moreover, the drug-specificity assays were performed for the *∆msm0031* deletion strain. The deletion strain exhibited similar killing patterns to the transposon mutant (Supplementary Materials, Figure S5). When we complemented both the transposon mutant and deletion strain, we could not retain the wild-type phenotype (Supplementary Materials, Figures S2, S6 and S7).

Next, we observed and quantified INH-killing kinetics of the *msm0031* transposon mutant and wild-type cells at micro scale using a microfluidic device. The main advantages of using a microfluidic-microscopy system in our experiments were the ability to track single cells from pre-antibiotic to post-antibiotic periods, to perfuse continuous medium and drug (which excluded drug degradation and waste accumulation), and to quantify morphology, lysis and division of the cells in real-time. Our microfluidic assays showed that our hypothesis based on bulk assays, which was that enhanced INH-killing of the *msm0031* transposon mutant was due to elevated lysis of the cells in the presence of INH, was wrong. The *msm0031* transposon mutant formed fewer colonies on standard LB plates compared to wild-type cells during the INH exposure in the batch culture. Therefore, as a consequence of the microfluidic assay, our prediction was increased cell lysis and a decreased number of intact cells for the *msm0031* transposon mutant. However, quantitative single-cell analysis presented contradictory results. It showed that upon INH-treatment, 24% of the PBP mutant cells were intact and PI negative, whereas this subpopulation was only 4.6% of the wild-type cells. In addition, both the division and lysis kinetics of the PBP transposon mutant and wild-type cells were quite different (Figure 5). The killing events of the PBP mutant between 40–48 h might be due to the tolerance of the cell wall, which cannot balance the osmatic pressure and, hence, most of the cells burst. Actually, the batch culture-killing curve also shows rapid killing around 30–48 h, as shown in Figure 3. The wild-type cells lysed more than the PBP transposon mutant and its cumulative lysis was more than its cumulative division. Hence, the wild-type cells were rapidly killed after 12 h of drug exposure in the microfluidic assay (32 h), which is also consistent with the batch-culture INH-killing profile. On the other hand, the *msm0031* transposon mutant actively divided up to 52 h and almost stopped its division at 60 h. Furthermore, it lysed less than wild-type cells. Finally, when we removed INH, 13.7% of the wild-type cells and 0.5% of the PBP mutant cells resumed growth. The subpopulation of the PBP mutant, which cannot form colonies, recovered growth but remained intact and PI-negative. It might be viable and non-culturable cell populations that could actively grow when specific culturing conditions were provided. The *msm0031* transposon mutant cells have always followed the same trends for the INH-killing profile, although the maximum lysis of the PBP mutant cells has varied within the 30–48 h of INH exposure.

The possible explanation for impaired colony formation and enhanced killing rate of the *msm0031::Tn* mutant could be explained by PbpA, since PbpA enrols at the final stage of the peptidoglycan synthesis. Another possible explanation might be that the *msm0031* gene is a part of a conserved gene cluster that is involved in coordinated regulation of cell division and/or cell elongation, and that interruption of any of these genes alters phenotypes of the cells (Supplementary Materials, Figures S1–S3).

To the best of our knowledge, our study is one of the first that combines culture-based assay results with on-chip-microscopy assays in regards to the drug killing aspects of the *M. smegmatis* transposon mutant, *msm0031*. In order to advance our knowledge about mechanisms of drug actions and functions of genes, we could incorporate the results obtained using different techniques at different scales, such as culture-based, population-level and microfluidic-microscopy-based, single-cell level assays. The results we obtained are the missing pieces of the puzzle of understanding how individuality of cells plays a role in the antibiotic response of the population, especially regarding the phenomenon of natural antibiotic resistance. 

## Figures and Tables

**Table 1:** Fractional survival ratio (FSR) for the *msm0031* transposon mutant and wild-type *M. smegmatis* cells (*** *p* < 0.0001, ** *p* < 0.01, * *p* < 0.05).

Antibiotics	FSR ± SE WT	FSR ± SE *msm0031*
**INH** (50 µg/mL)	1 ± 0.160	0.024 ± 0.040 ***
**EMB** (5 µg/mL)	1 ± 0.630	0.028 ± 0.050 **
**RIF** (200 µg/mL)	1 ± 0.001	1.670 ± 0.003
**ETH** (200 µg/mL)	1 ± 3.000	0.040 ± 3.700 *

## Supplementary Materials

The following are available online at http://www.mdpi.com/2072-666X/9/11/561/s1. Figure S1: mRNA expressions for *M. smegmatis msm0031* transposon mutant. Bars represent the ratio of mRNA levels relative to sigA, a constitutive gene whose expression remains relatively unchanged. Number of transcript copies determined by qRT-PCR using gene-specific primers as listed below. Figure S2: Chromosomal locus encoding the *msm0031* gene in *M. smegmatis*. Location of the transposon insertion indicated by vertical arrow. Figure S3: Confirmation for the *msm0031* deletion mutant measuring the mRNA expression levels. Bars represent the ratio of mRNA levels relative to sigA, a constitutive gene whose expression remains relatively unchanged. Number of transcript copies determined by qRT-PCR using gene specific primers as listed in materials and methods. The RT-qPCR experiments were performed at least 2 times. Figure S4: Deletion of *msm0031 gene altered morphology and growth rate. Deletion of msm0031* gene affected colony morphology (A), limited growth in standard 7H9 broth (B). Bright field images showed PBPA-inactivated cells were longer (C). Fluorescence microscopy of *msm0031* deletion mutant transformed with *Wag31–GFP* showed defective septum formation in the presence of INH (D). Using time-lapse movies generation time was calculated as 3 hours for the *msm0031* deletion cells (E). The measured cell area for the *msm0031* deletion strain was 2 times larger than WT (F). Figure S5: Batch culture drug-killing assays for the *msm0031* deletion mutant. WT *M. smegmatis* and the *msm0031* deletion mutant were treated with INH-50 µg/mL (A), ETH-200 µg/mL (B), EMB-5 µg/mL (C), and RIF-200 µg/mL (D). Serial dilutions of cultures were plated at the indicated time points to determine the CFU count. INH and EMB results are the mean ± standard error from 3 independent cultures; ETH and RIF results are the mean ± standard error from 3 independent cultures. Figure S6: Growth curves for the complemented *msm0031* transposon and deletion strains. Growth was monitored by measuring culture turbidity (OD_600_ nm) at the indicated time points. Figure S7: Batch culture drug-killing assays for the complemented *msm0031* deletion and transposon strains. WT *M. smegmatis*, the complemented *msm0031* deletion and complemented *msm0031* transposon mutant were treated with INH-50 µg/mL (A), ETH-200 µg/mL, (B), EMB-5 µg/mL (C), and RIF-200 µg/mL (D). Serial dilutions of cultures were plated at the indicated time points to determine the Log CFU. The results are representatives at least two independent experiments. Video S1: PBP mutant.
