# Supplementary material for: On-Chip Isoniazid Exposure of Mycobacterium smegmatis Penicillin-Binding Protein (PBP) Mutant Using Time-Lapse Fluorescent Microscopy
Source: Micromachines (Basel). 2018 Oct 31;9(11):561. doi: 10.3390/mi9110561 (PMC6266593; doi:10.3390/mi9110561)
Supplement: Supplementary file 1 [file micromachines-09-00561-s001.pdf]

Supplementary Materials: On-Chip Isoniazid Exposure of *Mycobacterium smegmatis* Penicillin-Binding Protein (PBP) Mutant Using Time-Lapse Fluorescent Microscopy

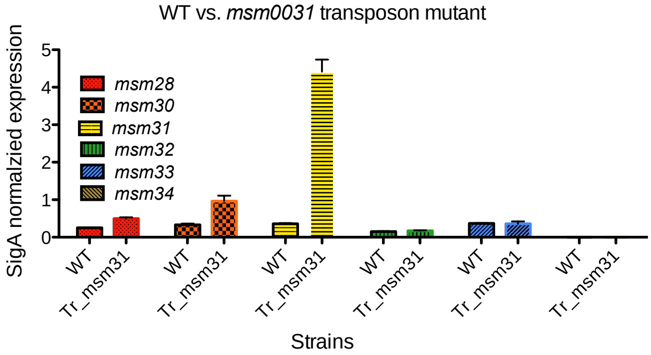

Figure S1. mRNA expressions for *M. smegmatis msm0031* transposon mutant. Bars represent the ratio of mRNA levels relative to sigA, a constitutive gene whose expression remains relatively unchanged. Number of transcript copies determined by qRT-PCR using gene-specific primers as listed below.

Complementation of the *msm0031::Tn* mutant. For complementation of the *msm0031::Tn* transposon mutant, the *msm0031* gene was PCR-amplified from wild-type *M. smegmatis* genomic DNA (Figure S1). The full-length gene was cloned into integrating plasmid as described below.

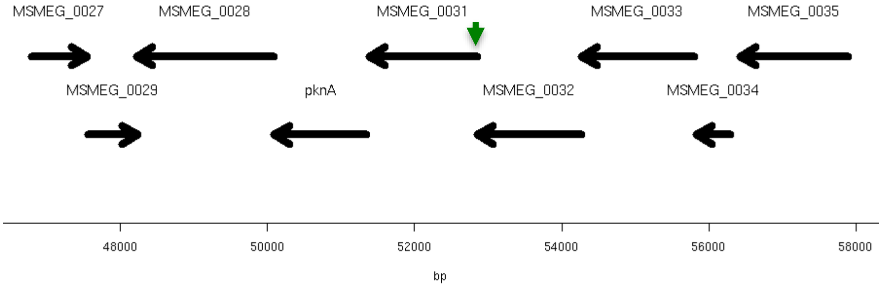

Figure S2. Chromosomal locus encoding the *msm0031* gene in *M. smegmatis.* Location of the transposon insertion indicated by vertical arrow.

Integrative complementation *(pND200_Strep_msm0031).* Complementation of the *msm0031::Tn* mutant with a single-copy attB-integrating plasmid (*pND200_Strep*) containing the intact *msm0031* gene was performed. The INH-mediated killing response of the *pND200_Strep_msm0031* strain followed wild-type kinetics only for the first 24 hours of drug exposure; thereafter, the killing kinetics were slower than wild-type (Figure S2). The minimum inhibitory concentrations for INH of the integrating complemented and over-expression strains were higher than 100 µg/mL (compared to 5 µg/mL for wild-type bacteria).

The *msm0031* deletion mutant: In-frame and unmarked deletion of the *msm0031* gene in the WT background was made by allelic exchange, using the two-step counter-selection method (Pelicic et al., 1996). The RT-qPCR experiment confirmed that the *msm0031* gene in the deletion strain was not expressed, Figure S3. Moreover, deletion of this gene might have affected the downstream genes (*msm0028* and *msm0030*).

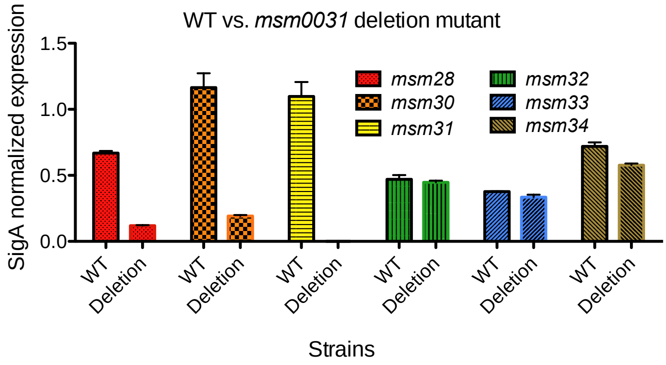

Figure S3. Confirmation for the *msm0031* deletion mutant measuring the mRNA expression levels. Bars represent the ratio of mRNA levels relative to sigA, a constitutive gene whose expression remains relatively unchanged. Number of transcript copies determined by qRT-PCR using gene specific primers as listed in materials and methods. The RT-qPCR experiments were performed at least 2 times.

Knockout of *msm0031* gene resulted in hindered growth and defective cell separation in *M. smegmatis* (Figure S4). The ability and time required to form colonies on standard LB plates was impaired relative to WT. This differs from the transposon mutant strain, which exhibited no growth phenotype and had higher transcript levels of *msm0028*, *msm0030*, and *msm0031*.

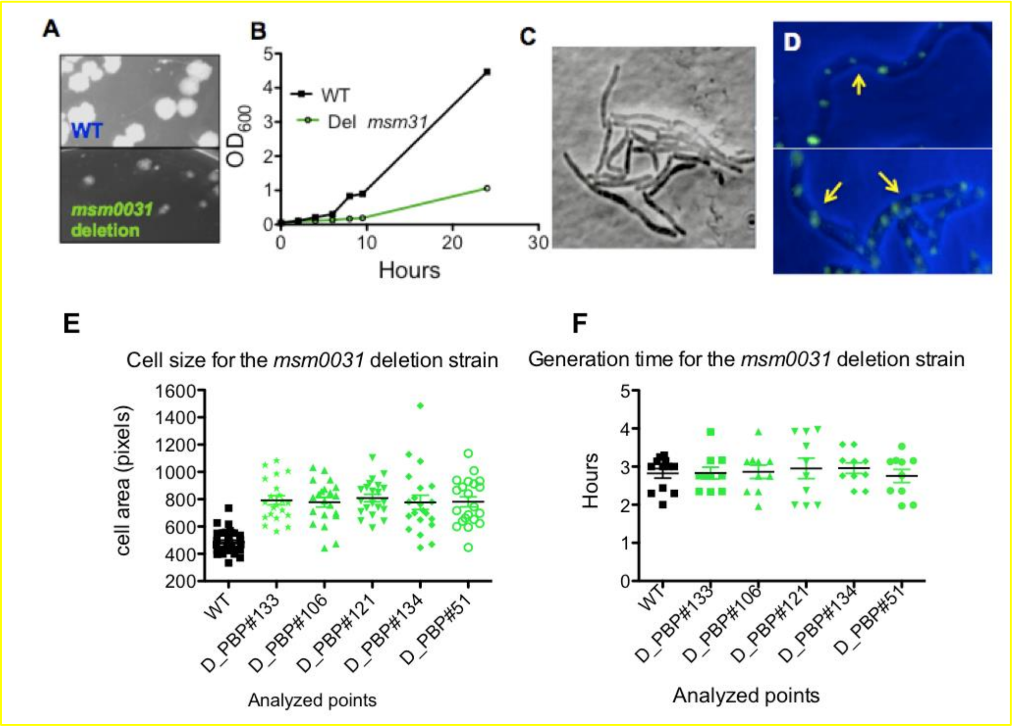

Figure S4. Deletion of *msm0031 gene altered morphology and growth rate. Deletion of msm0031* gene affected colony morphology (A), limited growth in standard 7H9 broth (B). Bright field images showed PBPA-inactivated cells were longer (C). Fluorescence microscopy of *msm0031* deletion mutant transformed with *Wag31–GFP* showed defective septum formation in the presence of INH (D). Using time-lapse movies generation time was calculated as 3 hours for the *msm0031* deletion cells (E). The measured cell area for the *msm0031* deletion strain was 2 times larger than WT (F).

Drug-Specificity for the *Δmsm0031* deletion strain. Experiments for drug susceptibility of the *Δmsm0031* deletion strain to INH, ETH, EMB and RIF resulted in quite similar killing patterns to *msm0031* transposon mutant. Both strains showed enhanced killing in the presence of INH, ETH and EMB. The *Δmsm0031* deletion strain was killed slightly more than the *msm0031::Tn* mutant, Figure

S5. The elongated cell shape might contribute to the enhanced killing profile because these cells might have colony-forming defect compared to transposon mutant.

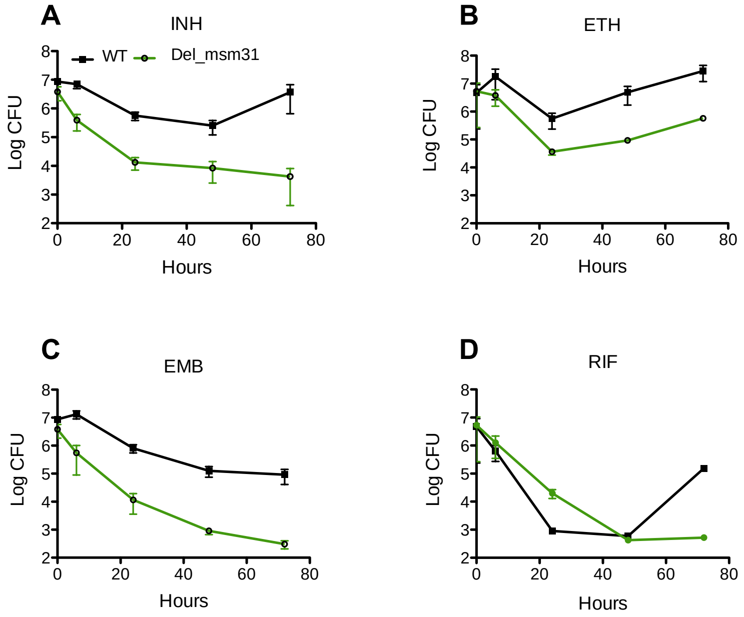

Figure S5. Batch culture drug-killing assays for the *msm0031* deletion mutant. WT *M. smegmatis* and the *msm0031* deletion mutant were treated with INH-50 µg/mL (A), ETH-200 µg/mL (B), EMB-5 µg/mL (C), and RIF-200 µg/mL (D). Serial dilutions of cultures were plated at the indicated time points to determine the CFU count. INH and EMB results are the mean ± standard error from 3 independent cultures; ETH and RIF results are the mean ± standard error from 3 independent cultures.

Complementation studies for the *Δmsm0031* and *msm0031::Tn* mutant strains. Complementation studies for the transposon mutant and the deletion strain were performed in parallel with the same construct. The wild-type copy of the *msm0031* gene was PCR-amplified from *M. smegmatis* genomic DNA and cloned into *pND200_Strep* integrating vector. Then, the *pND200_Strep_msm0031* plasmid was delivered into the *msm0031::Tn* and *Δmsm0031* strains, Figure S6.

The integrating complementation improved the growth defect for the complemented *msm0031* deletion strain. However, it was not possible to restore exact WT growth *[25]*. Delivery of the *pND200_Strep_msm0031* plasmid did not alter the growth kinetics of the complemented transposon mutant, which were like WT, Figure S7.

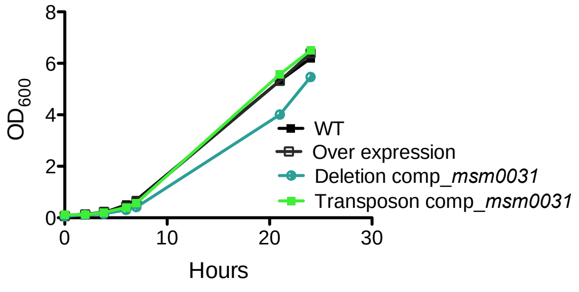

Figure S6. Growth curves for the complemented *msm0031* transposon and deletion strains. Growth was monitored by measuring culture turbidity (OD_{600} nm) at the indicated time points.

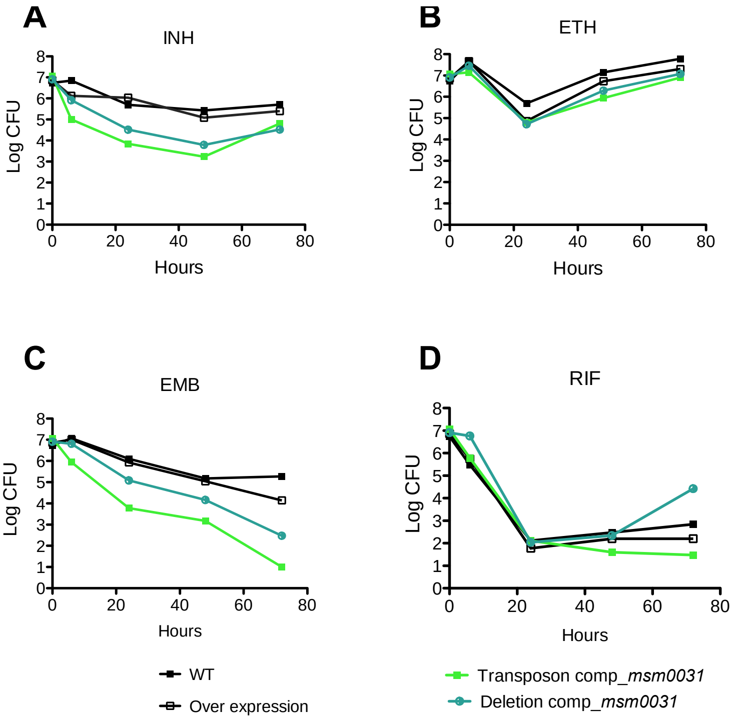

Figure S7. Batch culture drug-killing assays for the complemented *msm0031* deletion and transposon strains. WT *M. smegmatis,* the complemented *msm0031* deletion and complemented *msm0031* transposon mutant were treated with INH-50 μg/mL (A), ETH-200 μg/mL, (B), EMB-5 μg/mL (C), and RIF-200 μg/mL (D). Serial dilutions of cultures were plated at the indicated time points to determine the Log CFU. The results are representatives at least two independent experiments.

Besides, the 1% SDS response for the complemented strains was similar to WT. The obtained results showed that there was no killing difference for 6 and 24 hours (data not shown).

Primer used for sequencing transposon mutants:
Mycomarseq: CTT CTG AGC GGG ACT CTG GGG
Primers for complementation constructs:
C31F: GAT ATC TCA TGA ACC CTC CCG CAG C
C31R: GCT AGC ATG AAC ACC TCA CTG CGC CG
Primers for deletion constructs:
msm0031Up_F: GGT TAA TTA ACC TGG CGC TGT TCG CGG
msm0031Up_R: CCT AGG GGT GTT CAT ACC TTT TCG ATC ACC TCG G
msm0031Down_F: CCT AGG GGT TCA TGA GTC CGC GCA GG
msm0031Down_R: GGC GCG CCT CGG GCG CGA TGT ACT GCG C
Primers used to confirm the deletion of msm0031 gene:
M5B1L_F: CGT AGC CAA CGA CGC CCA
M5B1L_R: CGG TCT GGC AGG CGT TCT C
M5B1R_F: GTT GAT GAG CTC CAT CAC CAG GTA CG
M5B1R_R: TAC GCG TCG TTT CTG GTG CTG
Primers for qRT-PCR:
SigA_F: TCG ACT ACA CCA AGG GCT AC
msm0031_qPCR_F: CGG CTA CAC TAA CTG ACT TGA TGG T
msm0031_qPCR_R: CGG TTT TCG AAG CGA TCT
